# Perspective: Spectrin-Like Repeats in Dystrophin Have Unique Binding Preferences for Syntrophin Adaptors That Explain the Mystery of How nNOSμ Localizes to the Sarcolemma

**DOI:** 10.3389/fphys.2018.01369

**Published:** 2018-10-08

**Authors:** Justin M. Percival

**Affiliations:** Department of Molecular and Cellular Pharmacology, University of Miami Miller School of Medicine, Miami, FL, United States

**Keywords:** Duchenne muscular dystrophy, dystrophin, spectrin-like repeat, syntrophin, nNOS

## Abstract

Dystrophin is a massive multi-domain protein composed of specialized amino and carboxyl termini that are separated by 24 spectrin-like repeats. Dystrophin performs critical structural and signaling roles that are indispensable for the functional integrity of skeletal muscle. Indeed, the loss of dystrophin protein expression causes the muscle wasting disease, Duchenne muscular dystrophy (DMD). Substantial progress has been made in defining the functions of the domains of dystrophin, which has proven invaluable for the development of miniaturized dystrophin gene and exon skipping therapies for DMD. However, a long-standing mystery regarding dystrophin function is how dystrophin, and its adaptor and neuronal nitric oxide synthase mu (nNOSμ) binding partner α-syntrophin, cooperate to localize nNOSμ to the sarcolemma. Only when localized to the sarcolemma can nNOSμ override sympathetic vasoconstriction and prevent functional ischemia in contracting muscles. Current evidence suggests that spectrin-like repeat 17 of dystrophin and α-syntrophin cooperate to localize nNOSμ to the sarcolemma. However, the exact mechanism remains unclear and controversial because of equivocal evidence for direct binding of dystrophin and nNOSμ. Recently, an important study identified a novel α-syntrophin binding site within spectrin-like repeat 17, leading to a new model whereby α-syntrophin recruits nNOSμ to the sarcolemmal dystrophin complex by binding spectrin-like repeat 17. This model finally appears to solve the mystery of the dual requirement for dystrophin and α-syntrophin for sarcolemmal nNOSμ localization. The aim of the current perspective is to highlight this major advance in understanding of dystrophin’s role in localizing nNOSμ and its implications for current trials.

A long-standing mystery in the Duchenne muscular dystrophy (DMD) field is how the spectrin-like repeats of dystrophin, and the adaptor protein α-syntrophin cooperate to localize neuronal nitric oxide synthase mu (nNOSμ) to the sarcolemma. The translational importance of this mystery is underscored by pre-clinical studies indicating that the most therapeutic dystrophin-based gene therapy localized nNOSμ to the sarcolemma and by ongoing clinical trials of the therapeutic efficacy of miniaturized dystrophins that can or cannot restore sarcolemmal nNOSμ (NCT03375164, NCT03368742). Despite recent advances, the current model for how nNOSμ localizes to the sarcolemma is incomplete and controversial (Allen et al., 2016). The model is incomplete because we do not fully understand the requirement for the nNOSμ binding partner α-syntrophin. The model is controversial because of equivocal evidence for the ability of dystrophin to bind nNOSμ directly (Lai et al., 2013). A recent study by Adams et al. (2018), suggests a new model that may finally solve the mystery of the dual requirement for dystrophin and α-syntrophin for nNOSμ localization to the sarcolemma (**Figure 1**). The purpose of this commentary is to highlight this new model for sarcolemmal nNOSμ localization and to discuss its implications in the context of ongoing clinical testing of the ability of miniaturized dystrophins to mitigate dystrophic pathology in individuals with DMD.

**FIGURE 1:**
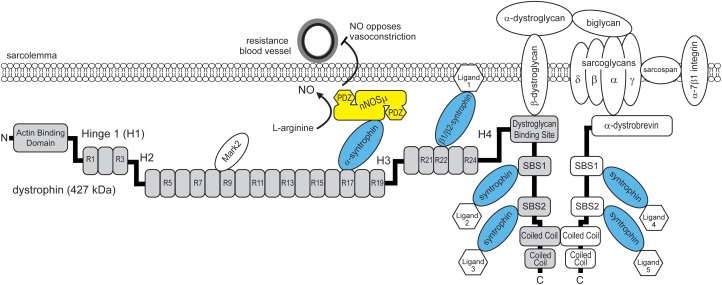
Model for the dual requirement of spectrin-like repeat 17 of dystrophin and α-syntrophin for nNOSμ localization to the sarcolemma. Dystrophin (gray boxes) contains SBS in spectrin-like repeat 17, spectrin-like repeat 22 and in its carboxyl terminus. The SBS in spectrin repeat 17 binds α-syntrophin that in turn recruits nNOSμ to the dystrophin protein complex. Sarcolemmal localization enables nNOSμ to oppose local sympathetic vasoconstriction and promote blood delivery to working muscles. nNOSμ has two PDZ domains that may recruit additional proteins to the sarcolemma. α-dystrobrevin contains two SBS. α and β syntrophins can recruit up to six unique ligands to the dystrophin protein complex. H1-H4: hinge region 1-4. R1-R24: spectrin-like repeat 1-24. Mark2: microtubule affinity regulating kinase 2. NO: nitric oxide.

In skeletal muscle, 427 kDa dystrophin is required for the sarcolemmal localization and function of many signaling proteins including cation and water channels, kinases and perhaps most famously, nNOSμ (Allen et al., 2016). nNOSμ is a flavoenzyme that oxidizes L-arginine to nitric oxide (NO) that is found in equivalent amounts at the sarcolemmal and cytosol in mature murine myofibers, and together with nNOSμ synthesizes the majority of NO in mature skeletal muscle cells. Loss of nNOS function in humans and mice leads to early mortality and is associated with severe achalasia, autism, and myopathy (Gyurko et al., 2002; Percival et al., 2010; Shteyer et al., 2015).

nNOSμ function is severely impaired in the dystrophin-deficient skeletal muscles of mdx mice and individuals with DMD. This is because dystrophin deficiency leads to a reduction in nNOSμ expression and prevents sarcolemmal localization. The loss of sarcolemmal nNOSμ function leads to unopposed sympathetic vasoconstriction which impairs local blood delivery to working muscles, and reduces muscle’s resistance to fatigue and lengthening contraction-induced injury (Thomas et al., 2003; Rebolledo et al., 2016). Sarcolemmal nNOSμ opposes local sympathetic vasoconstriction by inhibiting α-adrenergic receptors in contracting skeletal muscles (Thomas et al., 2003). In this way, sarcolemmal nNOSμ ensures optimal skeletal muscle perfusion during exercise in a protective process called functional sympatholysis. The precise function of functional sympatholysis is unknown, but it does not appear to play a major role in skeletal muscle strength or fatigue resistance, and likely facilitates the movement of energy substrates, metabolites, and myokines into and out of myocytes (Percival et al., 2010). In structurally compromised dystrophin-deficient skeletal muscle, repeated bouts of ischemia resulting from the loss of sarcolemmal nNOSμ may contribute to dystrophic pathogenesis by worsening contraction-induced muscle damage and inflammation (Asai et al., 2007; Lai et al., 2009). Indeed, the loss of nNOS promotes macrophage-driven inflammation exacerbating muscle damage (Froehner et al., 2014; Tidball and Wehling-Henricks, 2014). Taken together, these findings suggest important roles for nNOSμ in skeletal muscle perfusion, inflammation and contractile performance, and highlight the significance of understanding nNOSμ regulation, particularly the mechanisms governing the sarcolemmal localization of nNOSμ.

Early studies of dystrophin-deficient skeletal muscles from mice and patients firmly established that dystrophin was required for normal nNOSμ expression and sarcolemmal localization (Brenman et al., 1995). The carboxyl terminus of dystrophin contained two binding sites for the adaptor α-syntrophin whose PDZ domain binds a β-hairpin loop within nNOSμ. This leaves the two PDZ domains of the nNOSμ heterodimer free to interact with other proteins. Biochemical, genetic, and x-ray crystallography studies from many groups all agreed that α-syntrophin directly bound nNOSμ and was the best candidate to mediate the interaction of dystrophin and nNOSμ (Brenman et al., 1996; Hillier et al., 1999). Accordingly, nNOSμ could not localize to the sarcolemma in mice lacking α-syntrophin expression or in mice expressing a mutant α-syntrophin lacking its PDZ domain (Adams et al., 2001). These findings confirmed biochemical analyses by demonstrating the specific *in vivo* requirement of the PDZ domain of α-syntrophin for the sarcolemmal targeting of nNOSμ.

However, studies in mdx mice and Becker muscular dystrophy patients that expressed a variety of truncated dystrophin proteins indicated that nNOSμ and α-syntrophin could associate with the sarcolemma independently of the two syntrophin binding sites (SBS) in dystrophin’s carboxyl terminus (**Figure 1**). Furthermore, the data showed that sarcolemma-localized α-syntrophin was necessary, but not sufficient for nNOSμ association with the sarcolemma. These studies highlighted the importance of spectrin-like repeat sequences around exons 42–47 in the central rod domain of dystrophin, for the targeting of sarcolemmal nNOSμ (Brenman et al., 1995; Chao et al., 1996). However, nNOSμ could not be shown to interact directly with the spectrin-like repeat sequences of dystrophin. Affinity chromatography analyses of whole muscle skeletal muscle extracts showed that nNOSμ could only bind α-syntrophin, but not dystrophin (Chao et al., 1996).

Recently, the sequence within dystrophin required for sarcolemmal nNOSμ localization was narrowed down to a 10 amino acid microdomain within spectrin-like repeat 17 encoded by exon 45 (Lai et al., 2013). *In vivo* transfection of mdx muscles with mutant dystrophins lacking the carboxyl terminus α-SBS and spectrin-like repeat 17 clearly demonstrated that spectrin-like repeat 17 was required for nNOSμ sarcolemmal localization. In contrast to earlier studies, Lai et al. (2013) concluded that nNOSμ could indeed bind dystrophin at the 10 amino acid microdomain of spectrin-like repeat 17 based on positive interactions in the yeast-2 hybrid system. However, spectrin-like repeats 16 and 17 contain coiled-coil secondary structures that are notorious for generating false positive interactions in yeast-2-hybrid assays. In addition, the yeast-2-hybrid data conflicted with earlier affinity chromatography studies in skeletal muscle homogenates that argued against a direct interaction between nNOSμ and dystrophin. These results raised questions about the interaction of dystrophin and nNOSμ, and most importantly did not explain the mystery of the dual requirement of spectrin-like repeat 17 of dystrophin and α-syntrophin for nNOSμ localization to the sarcolemma.

The recent study by Adams et al. (2018) may finally provide the answer to this mystery. They performed a search for novel SBS in dystrophin, focusing on dystrophin’s spectrin-like repeat region and amino terminus. Skeletal muscle syntrophins (α1, β1, and β2) are important adaptor proteins for dystrophin, and dystrophin-related proteins including α-dystrobrevin, which also contain multiple SBS (**Figure 1**; Allen et al., 2016). Syntrophins bind a large variety of ligands including cation and water channels, tyrosine kinase receptors, kinases, phosphatases, and other enzymes including nNOSμ (Allen et al., 2016). Therefore, a major function of syntrophins is to recruit signaling proteins to the dystrophin protein complex. To identify novel binding sites, Adams et al. (2018) identified a new SBS consensus sequence using known SBS in dystrophin and related proteins from evolutionarily close and distant species. The SBS consensus sequence was a 14 amino acid long α-helix with a strictly conserved histidine at position zero. Using this SBS consensus sequence, they identified three new potential SBS in human dystrophin: one in spectrin-like repeat 22, one in spectrin-like repeat 23, and importantly one in spectrin-like repeat 17. Remarkably, the newly identified SBS in spectrin-like repeat 17 contained the entire 10 amino acid microdomain necessary for sarcolemmal nNOSμ localization (Lai et al., 2013). This suggested that the 10 amino acid microdomain within spectrin-like repeat 17 was in fact part of a novel SBS.

Adams et al. (2018) then demonstrated that peptides containing the putative SBS in spectrin-like repeats 17, 22, but not 23, could pull down syntrophin from skeletal muscle homogenates. Importantly, spectrin-like repeats 17 and 22 had unique syntrophin isoform preferences. The SBS of spectrin-like repeat 17 bound only α-syntrophin, while the SBS of spectrin-like repeat 22 bound β1-syntrophin and β2-syntrophin (**Figure 1**). The preference may be caused by several differences in charged amino acid identity within the two SBS. α- and β-syntrophin bind different ligands, for example, α-syntrophin binds nNOSμ, while β2-syntrophins bind the microtubule-associated serine/threonine kinase 2 (Mast2) that regulates microtubules and cell survival (Allen et al., 2016). Therefore, these findings suggested that spectrin-like repeats 17 and 22 regulate the interactions of specific syntrophin isoforms (and their ligands) with dystrophin, and contribute to the diverse signaling functions of the dystrophin protein complex.

Having shown that the SBS within spectrin-like repeat 17 bound α-syntrophin *in vitro*, Adams et al. (2018) then tested if α-syntrophin bound at spectrin-like repeat 17 was necessary for sarcolemmal nNOSμ localization *in vivo*. As a control, they expressed a microdystrophin containing spectrin-like repeats 16 and 17 that expectedly restored sarcolemmal nNOSμ localization in dystrophin-deficient muscles of mdx mice. However, this microdystrophin could not restore sarcolemmal nNOSμ localization in dystrophin-α-syntrophin double knockout mice whose skeletal muscles lacked both dystrophin and α-syntrophin. This result demonstrated that the localization of nNOSμ to the sarcolemma required both spectrin-like repeat 16/17 and α-syntrophin, consistent with the model that α-syntrophin was recruiting nNOS to the sarcolemma by binding spectrin-like repeat 16/17.

As mentioned above, dystrophin has two α-SBS in its carboxyl terminus. In addition, dystrophin binds the related protein α-dystrobrevin, which also contains two α-SBS (**Figure 1**). Current evidence now questioned the importance of these sites for the localization of nNOSμ. To clarify the importance of these additional α-syntrophin sites for sarcolemmal nNOSμ localization, Adams et al. (2018), generated mdx mice (dCTmdxdd) whose skeletal muscles lacked α-dystrobrevin (dd) and the carboxyl terminus of dystrophin (dCT). dCTmdxdd mice lacked the α-SBS in α-dystrobrevin and carboxyl terminus of dystrophin, while retaining spectrin-like repeat 17 with its α-SBS. In dCTmdxdd mice α-syntrophin and nNOSμ could still be detected at the sarcolemma, although α-syntrophin labeling was reduced as expected. These findings support the model that there are multiple α-syntrophin sites in dystrophin and α-dystrobrevin that are required for full sarcolemmal association of α-syntrophin, but that only the α-SBS within spectrin like repeat 17 is required for sarcolemma nNOSμ localization. While these α-SBS are not required for the steady state targeting of nNOSμ to the sarcolemma, they likely provide additional sites for other α-syntrophin ligands to interact with the sarcolemmal dystrophin protein complex.

Taken together, the findings of Adams et al. (2018), suggest a new model where only the α-syntrophin SBS in spectrin-like repeat 17 is necessary for nNOSμ localization to the sarcolemma (**Figure 1**). This is because spectrin-like repeat 17 contains a SBS that allows the specific docking of α-syntrophin and its bound ligand nNOSμ. This model reconciles the dual requirement for spectrin-like repeat 17 of dystrophin and α-syntrophin and provides a compelling explanation for how α-syntrophin and dystrophin cooperate to localize nNOSμ to the sarcolemma.

Dystrophin’s spectrin-like repeats are often viewed as dispensable springs or shock absorbers (Allen et al., 2016). Recent studies, including the study of Adams et al. (2018), challenge this view because spectrin-like repeats have unique binding preferences for syntrophin isoforms and other proteins, including microtubule affinity regulating kinase 2 (Mark2), that customize the dystrophin protein complex for different biological functions. Spectrin-like repeat 17 is essential for localizing sarcolemmal nNOSμ, which is essential for the ability of nNOSμ to oppose sympathetic vasoconstriction and promote local blood delivery to working muscles (**Figure 1**). Another example comes from the finding that spectrin-like repeats 8/9 are necessary for normal Mark2 localization and function in satellite cell regeneration (Yamashita et al., 2010; Dumont et al., 2015). Although the identity and function of the ligands of β1 and β2 syntrophin bound at spectrin-like repeat 22 remain to be determined, they also represent new roles for spectrin-like repeats and therefore new functions for the dystrophin protein complex. Taken together, these findings suggest that spectrin-like repeats also facilitate the selective interaction of signaling proteins with dystrophin, thereby providing an exquisite degree of spatial control that ultimately enables the dystrophin complex to perform its diverse roles in fundamental biological processes in muscle.

Finally, like all good studies this one raises important new questions like “what is the mechanism underpinning the syntrophin binding preferences of spectrin-like repeats 17 and 22”, and “what is the functional significance of β syntrophins bound at spectrin-like repeat 22 and the other α SBS in dystrophin and α-dystrobrevin”. Nonetheless, the study of Adams et al. (2018) represents an important advance in the understanding the organization of the dystrophin protein complex and its customization for signaling roles. The identification of biologically significant spectrin-like repeat sequences may ultimately contribute to the further optimization of dystrophin-based gene and exon skipping therapies for DMD.

Indeed, it will soon be possible to compare the therapeutic effectiveness of microdystrophins that can or cannot restore sarcolemmal nNOSμ in individuals with DMD. An ongoing Phase 1/2a study (NCT03375164) of the therapeutic efficacy of a systemically delivered microdystrophin that lacks spectrin-like repeat 17 and the ability to restore sarcolemmal nNOSμ reported early promising results in three participants, including a marked reduction in serum creatine kinase, a biomarker of skeletal muscle damage. Similarly, the IGNITE DMD trial (NCT03368742) seeks to determine the therapeutic effectiveness of a comparably delivered microdystrophin that possesses spectrin-like repeat 17 and thus the ability to restore sarcolemmal nNOSμ. The IGNITE DMD trial is yet to report results, but a large body of preclinical evidence suggests that this microdystrophin will be most efficacious due to additional sarcolemmal nNOSμ-driven improvements in skeletal muscle blood delivery, inflammation and contractile performance. The results of these two trials will likely inform future decisions as to whether miniaturized dystrophins need to restore sarcolemmal nNOSμ for maximal therapeutic benefit in individuals with DMD. The findings of Adams et al. (2018) provide us with an improved blueprint of how sarcolemmal nNOSμ restoration can be achieved.

## Author Contributions

JP conceptualized and wrote the article.

## Conflict of Interest Statement

The author declares that the research was conducted in the absence of any commercial or financial relationships that could be construed as a potential conflict of interest.
